# Antibody–drug conjugates in solid tumors: a look into novel targets

**DOI:** 10.1186/s13045-021-01035-z

**Published:** 2021-01-28

**Authors:** Carmen Criscitiello, Stefania Morganti, Giuseppe Curigliano

**Affiliations:** 1 Division of Early Drug Development for Innovative Therapy, European Institute of Oncology, IRCCS, Via Ripamonti 435, 20141 Milan, Italy; 2 Department of Oncology and Haematology (DIPO), University of Milan, Milan, Italy

**Keywords:** Solid tumors, Antibody–drug conjugates, Cancer, ADCs

## Abstract

Antibody–drug conjugates (ADCs) are a relatively new class of anticancer agents designed to merge the selectivity of monoclonal antibodies with cell killing properties of chemotherapy. They are commonly described as the “Trojan Horses” of therapeutic armamentarium, because of their capability of directly conveying cytotoxic drug (payloads) into the tumor space, thus transforming chemotherapy into a targeted agent. Three novel ADCs have been recently approved, i.e., trastuzumab deruxtecan, sacituzumab govitecan and enfortumab vedotin, respectively, targeting HER2, Trop2 and Nectin4. Thanks to progressive advances in engineering technologies these drugs rely on, the spectrum of diseases sensitive to these drugs as well as their indications are in continuous expansion. Several novel ADCs are under evaluation, exploring new potential targets along with innovative payloads. This review aims at providing a summary of the technology behind these compounds and at presenting the latest ADCs approved in solid tumors, as well as at describing novel targets for ADCs under investigation and new strategies to optimize their efficacy in solid tumors.

## Background

Antibody–drug conjugates (ADCs) are complex targeted agents composed by a cytotoxic drug hanging on an antibody scaffold. Upon binding with the cell surface antigen targeted by the specific antibody, the ADC is internalized by the tumor cell and processed by the endo-lysosomal system. The linker that connects payload and antibody is then cleaved, and the payload released into the cytoplasm, where it finally induces cell apoptosis via its cytotoxic pathway. Beyond this “classical” pathway, ADCs can induce tumor cell death through the so-called bystander effect, which occurs when the cytotoxic warhead diffuses across the cell membrane to neighboring cells, thus inducing their apoptosis [1].

Indeed, three key elements define an ADC: the antibody directed against a specific tumor antigen, the cytotoxic drug (called payload or warhead) and the linker connecting the payload to the antibody [2].

### Target

Ideal targets of ADCs are antigen expressed exclusively on the surface of tumor cells. Lineage-specific antigens expressed by hematological malignancies have been thereby extensively explored as perfect candidates with many specific ADCs already approved and other under development [3]. Unfortunately, the concept of lineage-specificity does not apply to solid tumors, for which antigens expressed are mainly “tumor-associated” instead of “tumor-specific”, i.e., expressed on tumor cells but also on normal cells (weakly or limited to a given tissue type). This implies both a share of on-target/off-tumor toxicity for all these compounds dependent on expression of the specific target by normal cells and a reduced amount of intratumoral drug delivery [4].

### Antibody

Antibodies incorporated into ADCs are mainly humanized antibodies, significantly less immunogenic than murine and chimeric monoclonal antibodies (mAb) [2]. Most of them are based on the IgG1 isotype, preferred over the IgG2 and IgG4 because of its easier production [5]. The IgG1 isotype also retains higher immunogenic functions supporting both antibody-dependent cellular cytotoxicity (ADCC) and complement-dependent cytotoxicity (CDC) reactions [2]. Interestingly, more recent ADCs were specifically designed to loss these immunogenic properties considered too toxic if paired to potent warheads. Indeed, some companies preferred IgG2 or IgG4 isotypes, while others applied Fc-mutated variants of IgG1 isotype [2].

### Linker

Linkers are biochemical compounds connecting the antibody to the payload. ﻿Effective linkers have to guarantee ADC stability in the bloodstream as well as to allow an efficient cleavage upon internalization into tumor cells. Linkers can be classified into cleavable and non-cleavable according to their chemical properties [2, 6]. ﻿Non-cleavable linkers consist of stable bonds resistant to proteolytic degradation, so that cleavage occurs only after lysosome internalization and complete degradation of the antibody. These linkers have higher stability than cleavable ones, but can suffer from lower membrane permeability. Conversely, cleavage of cleavable linkers can depend on external pH (acid-labile linkers), specific lysosomal proteases (protease-cleavable linkers) or glutathione reduction (disulphide linkers) [2, 6–8]. All aspects of ADC pharmacology can be influenced by the specific design of the linker, as drug stability into circulation, tumor cell permeability, drug-to-antibody ratio (DAR, i.e. the number of payload moleculas carried by each antibody), and extent of the bystander effect [2, 7].

### Payload

Most of cytotoxic payloads developed belong to two major families: tubulin inhibitors (﻿maytansinoids or auristatins) and DNA-damaging agents (mainly calicheamicins) [9]. All of them are extremely potent cytotoxic drugs characterized by an IC50 (the inhibitory concentrations that inhibited 50% of cells) in the nanomolar and picomolar range, and an unfavorable toxicity profile if administered systemically [3]. Most of these drugs were discovered decades ago, but their development discontinued prematurely because of a narrow therapeutic index. Conversely, conjugation into ADC hides the cytotoxic drug in the bloodstream to convey it directly into tumor cells, thus significantly reducing toxicity of these potent agents. Oppositely, drugs with a more favorable therapeutic index such as anthracyclines or taxoids failed their development as warheads, being the IC50 too low to be effective when released in a small amount into tumor cells [2].

Calicheamicins are actinomycete-derived antibiotics that binds to the DNA minor groove and finally cause cell death by cleaving the double-stranded DNA. ﻿Ozogamicin is the most developed payload of this class, incorporated into two ADCs developed for hematological malignancies: ﻿gemtuzumab ozogamicin, an anti-CD33 ADC approved for acute myeloid leukemia, and inotuzumab ozogamicin, ﻿a recently approved anti-CD22 ADC against acute lymphoblastic leukaemia [10].

Anti-microtubules agents represent the large majority of warheads developed so far, mainly because of their favorable biochemical properties. Auristatins, like monomethyl auristatin E (MMAE) and F (MMAF), are synthetic compounds derived from dolastatin 10, a natural antimitotic drug [11]. Brentuximab vedotin, ﻿polatuzumab vedotin and ﻿enfortumab vedotin are all approved ADCs carrying the MMAE payload. The shared term “vedotin” here refers to MMAE plus its linking structure, according to the nomenclature defined by the World Health Organization’s International Nonproprietary Names (INN) for pharmaceuticals. Maytansinoids (DM1 and DM4) are synthetic derivatives of maytansine that also act by inhibiting microtubule polymerization [12]. DM1 is the warhead carried by trastuzumab emtansine (T-DM1), the first ADC approved for solid tumors.

Most of ADCs under investigation harbor such microtubule inhibitors, but many of them recently failed their clinical development because of dose-limiting toxicities (DLTs). Latest researches focused on less cytotoxic drugs, with a lower IC50, but whose activity could have been enhanced exploiting different strategies such as an increased DAR or the bystander effect. Among them, the most promising new class of payload is represented by camptothecins, a well-known class of anticancer agents targeting topoisomerase I (TOP1) [13]. Sacituzumab govitecan is a recently approved ADC conjugating an anti-Trop2 antibody with SN-38, the active metabolite of irinotecan. SN-38 is nearly 1000 times more active than irinotecan and cannot be administered as unbound drug because of its toxicity and poor solubility [2]. Deruxtecan (DX-d) is another payload with anti-TOP1 activity that recently reached the therapeutic armamentarium with the anti-HER2 trastuzumab deruxtecan (T-DXd) and already approved for breast cancer and under development for several tumor types.

## Established targets with approved ADCs

Four ADCs are currently FDA-approved for the treatment of solid tumors: trastuzumab emtansine and trastuzumab deruxtecan, both anti-HER2; enfortumab vedotin, targeting Nectin-4; and sacituzumab govitecan, active against Trop2 (Table 1).

**Table 1:** FDA-approved ADCs for the treatment of solid tumors

	Target	Payload	Linker	DAR	Indications
Trastuzumab emtansine (T-DM1)	HER2	Maytansine (microtubule inhibitor)	Non-cleavable thioether linker	3.5	HER2-positive mBC pretreated with trastuzumab and a taxane; HER2-positive early BC with residual disease after neoadjuvant trastuzumab and taxanes
Trastuzumab deruxtecan (T-DXd)	HER2	Deruxtecan (topoisomerase inhibitor)	Protease-cleavable linker	8	HER2-positive mBC progressing to two or more prior anti-HER2-based regimens in the metastatic setting
Enfortumab vedotin (EV)	Nectin-4	Monomethyl auristatin E (microtubule inhibitor)	Protease-cleavable linker	3.8	Locally advanced/metastatic UC previously treated with a PD-(L)1 and a platinum-based chemotherapy in the (neo)adjuvant or metastatic setting
Sacituzumab govitecan (SG)	Trop-2	SN-38 (topoisomerase inhibitor)	Hydrolysable linker	7.5	Triple-negative mBC (who have received at least two prior therapies for metastatic disease)

### HER2

HER-2 is a transmembrane growth factor receptor belonging to the HER family proteins, along with HER1 (EGFR), HER3 and HER4. Several EGF ligands have been described, even if none is specific to HER2. The binding of any of them to HER1, HER3 or HER4 induce receptor dimerization and the subsequent activation of the tyrosine-kinase intracellular domain, triggering many different downstream pathways. Among all HER proteins, HER2 is the preferred dimer partner [14].

HER2 is widely expressed on the surface of many adult tissues, even if its role has been primarily associated to organogenesis [15]. The role of HER2 as oncogene is mainly related to gene overexpression, which increases the number of HER2 heterodimers on cell surface and induces both cell transformation and tumorigenic growth not only in breast cancer, but also in several other cancer types [16–18].

HER2 somatic mutations have also been described [19, 20]. In the 90% of cases, mutations occur in the extracellular or kinase domain, while the juxtamembrane (7%) or transmembrane domains (3%) are barely mutated. Given the rarity of these mutations, their role as oncogenic drivers has been questioned for many years, and only recent studies proved their tumorigenic role along with their sensitivity to anti-HER2 agents. Bladder cancer has the highest prevalence of HER2 mutations (9–18%), followed by uterine cervix (6%), colorectal (5.8%), lung (4%) and breast cancer (4%) [19].

#### Trastuzumab emtansine

T-DM1 is the first ADC approved in solid tumors. Trastuzumab, the anti-HER2 IgG1 mAb, is here linked by a thioether linker to emtansine, an inhibitor of microtubule polarization. Approval of T-DM1 in metastatic breast cancer (mBC) relies on evidence from the phase 3 EMILIA trial [21], investigating T-DM1 versus lapatinib plus capecitabine after a trastuzumab-based first line therapy, and the TH3RESA trial [22], comparing T-DM1 versus treatment of physician’s choice (TPC) in a more pretreated population. Both studies showed a significant improvement in progression-free survival (PFS) and overall survival (OS), with a good safety profile. Following results of the KATHERINE trial [23], T-DM1 has been also approved in the post-neoadjuvant setting for patients with residual invasive BC after neoadjuvant therapy.

#### Trastuzumab deruxtecan

Trastuzumab deruxtecan (DS-8201a; T-DXd) is an anti-HER2 ADC in which trastuzumab is conjugated with DXd, a novel topoisomerase I inhibitor payload. It is approved in US and Japan for patients with advanced or metastatic HER2-positive BC after at least two prior anti-HER2-based regimens and is under accelerated assessment in Europe. In 2020, the Food and Drug Administration (FDA) granted a breakthrough therapy designation to T-DXd for the treatment of patients with metastatic, HER2-mutated non-small cell lung cancer (NSCLC) after a platinum-based therapy and priority review for the treatment of HER2-positive metastatic gastric or gastroesophageal junction adenocarcinoma.

Beyond the new potent warhead, many additional biochemical improvements differentiate T-DXd from previous anti-HER2 ADCs, justifying its activity across several cancer types. First, the DAR of T-DXd is twice that T-DM1 (8 vs 3–4), leading to a significantly increase of drug concentration in tumor cells [24]. Secondly, the link is made by a novel lysosome-cleavable peptide that is sensitive to enzymes commonly detected in tumor microenvironment, such as cathepsins [24]. Finally, the payload is permeable to cell membrane and can diffuse into adjacent cells. These two latter aspects maximize the bystander effect of T-DXd, making it effective also against formally HER2-negative cells [25]. In preclinical studies, T-DXd showed antitumor activity across a wide range of HER2-expressing tumor models [24].

Approval in mBC relies on data from 184 women with HER2-positive mBC enrolled in the phase II DESTINY-Breast01 study [26]. Despite being heavily pretreated (median number of previous lines of therapy for metastatic disease was 6, and all patients were pretreated with T-DM1), these patients achieved an overall response rate (ORR) of 60.3%, with a median duration of response (DOR) of 14.8 months. The safety analysis was conducted in a pooled population of 234 patients with BC, who received at least one dose of T-DXd at 5.4 mg/kg enrolled in the DESTINY-Breast01 trial and in the phase I DS8201-A-J101 study (data not published). The most common AEs (frequency ≥ 20%) were gastrointestinal disorders, hematological toxicities, alopecia and cough, while most frequent grade 3/4 AEs were neutropenia (20.7%), leukopenia (6.5%), lymphopenia (6.5%), anemia (8.7%), nausea (7.6%) and fatigue (6.0%). Remarkably, 13.6% of patients experienced an interstitial lung disease (ILD), with four reported fatal outcomes (2.2%). A subgroup analysis of the DESTINY-Breast01 showed that T-DXd is active in patients with brain metastasis, with an ORR of 58.3% and a median PFS of 18.1 months [27]. Of note, several studies are investigating T-DXd in patients with HER2-low tumors, defined as IHC1+ or IHC2+/FISH not amplified, taking advantage of the high bystander effect showed in preclinical studies [25].

Gastric and gastroesophageal junction (GC/GEJC) cancers are the only other malignancies showing a significant benefit with an anti-HER2 agent [28]. Nevertheless, HER2-targeted agents’ efficacy is far from being comparable to that observed in BC, and no HER2-directed drugs other than trastuzumab have been approved in these diseases. This gap has mainly been attributed to tumor heterogeneity of HER2-expression in GC/GEJC, eventual co-occurrence of HER2 amplification with other oncogenic mutations and to mechanisms of primary and secondary resistance to anti-HER2 treatments [29].

The DESTINY-Gastric01 trial investigated T-DXd versus chemotherapy of TPC in patients with HER2-positive GC progressing to at least 2 previous lines of treatment, including trastuzumab. The trial met its primary endpoint, with an ORR of 51% in patients in the T-DXd group, as compared to 14% in the control arm (*P* < 0.001). Of note, OS was significantly longer for patients receiving T-DXd (median OS, 12.5 vs 8.4 months) [30]. The high DAR ratio of T-DXd, the membrane permeability of deruxtecan and the efficient bystander effect may give account of the increased efficacy of trastuzumab deruxtecan in highly heterogeneous tumors as GC.

T-DXd is under investigation also in patients with HER2-positive NSCLC and colorectal cancers. Interim data from the phase II DESTINY-Lung01 showed promising clinical activity with high ORR (61.9%) and durable responses (median DOR not reached at data cutoff) [31], leading to the FDA breakthrough therapy designation for patients with HER2-mutated NSCLC. Similarly, the DESTINY-CRC01 showed significant antitumor activity in heavily pretreated patients with HER2-expressing colorectal cancers (ORR 45%), maintained in patients pretreated with anti-HER2 agents (ORR 43.8%) [32].

Several trials investigating T-DXd in different tumor types and/or in combination with other agents are ongoing.

#### SYD985

SYD985 (trastuzumab duocarmazine) is a novel ADC that conjugates trastuzumab with the alkylating agent duocarmycin, via a cleavable linker. As deruxtecan, duocarmycin is permeable to cell membranes, hence able to induce an effective bystander killing. After promising phase I data [33], the phase III TULIP trial (NCT03262935) is currently comparing SYD985 versus TPC in patients with HER2-positive mBC pretreated with at least 2 HER2-based regimens.

Noteworthy, many other anti-HER2 ADCs are under evaluation, including A166, XMT-1522, MEDI-4276, ARX788, RC48-ADC, BAT8001 and PF-06804103.

### NECTIN4

Nectin-4 is a type I transmembrane glycoprotein belonging to the Ig superfamily proteins, and as other Nectins are primarily involved in the formation and maintenance of adherence and tight junctions [34]. Nectin-4 is mainly expressed by embryo and fetal tissues, while its expression is low in normal adult tissues [35]. Conversely, it has been found to be overexpressed by several cancers, such as breast [36], lung [37] and ovarian [38] cancer and demonstrated to promote tumor growth and proliferation.

#### Enfortumab vedotin

In December 2019, enfortumab vedotin (EV) was granted accelerated approval by the FDA for treatment of patients with locally advanced or metastatic urothelial cancer (mUC) previously treated with a PD-(L)1 and a platinum-containing chemotherapy in the neoadjuvant/adjuvant or metastatic setting. EV is an ADC targeting nectin-4, consisting in a fully humanized IgG1 antibody conjugated to the microtubule inhibitor MMAE, throughout a protease-cleavable linker. Regulatory approval relies on preliminary data about antitumor activity from cohort 1 of the small EV-201 trial [39], which enrolled 125 patients with urothelial cancer previously treated with platinum-base chemotherapy and a checkpoint inhibitor. The observed ORR was 44%, with 12% of complete responses (CR). Median DOR was 7.6 months. These results were further supported by data from the EV-101 phase I study [40], which reported an ORR of 44.6% and a median DOR of 7.5 months from an analogous population. Notably, previously reported ORR and DOR of alternative therapies for mUC in this setting are significantly lower [41, 42].

Safety analysis was conducted on 310 patients from 3 different trials [43]. ﻿Most frequently reported toxicities of any grade were fatigue, peripheral neuropathy along with gastrointestinal and skin toxicities. Grade ≥ 3 AEs occurring in more than 5% of patients were rash, diarrhea, and fatigue. ﻿The FDA labels hyperglycemia, peripheral neuropathy, ocular disorders, skin reactions, infusion site extravasations and embryo-fetal toxicity as warnings and precautions. Many of these toxicities (hyperglycemia, skin rash and peripheral neuropathy) have been described also for other ADCs carrying MMAE and have been attributed mainly to the payload [44]. Skin toxicity instead may be at least partially classified as on-target/off-tumor toxicity, being nectin-4 highly expressed by cutaneous cells [43].

Nectin-4 expression was not requested for inclusion in abovementioned trials. Nevertheless, 120 patients enrolled in the EV-201 study had tissue available for analysis and nectin-4 expression was assessed by immunohistochemistry (IHC) using H-scores. Almost all patients had detectable nectin-4 expression, with a median H-score of 290 (range 14–130) [39]. Notably, response to EV was independent from H-score [43].

EV is under investigation in several tumor types, and the confirmatory phase III trial in mUC is ongoing (EV-301 study; NCT03474107).

### TROP-2

Trop-2 (trophoblast antigen 2) is a transmembrane glycoprotein coded by the gene TACSTD2, which primarily acts as intracellular calcium signal transducer [45]. Trop2 was firstly discovered in trophoblast cells, and under physiological conditions this protein is essential in both embryogenesis and fetal development [46]. Several normal tissues also express Trop2, including skin, uterus, bladder, esophagus, oral mucosa, nasopharynx and lungs. Several putative ligands have been described, including IGF-1, cyclin D1 and claudin-1 and -7. Trop2 is overexpressed in many epithelial tumors, including but not limited to breast, urothelial, lung, gynecological and gastro-intestinal carcinomas [47] and has been associated to poor prognosis. Its overexpression directly promotes tumor growth by inducing several different oncogenic pathways. Although many of them have been described, including the ERK/MAPK and the NF-kB pathways, the exact role of each of them in the specific tumor type has to be elucidated yet [48].

#### Sacituzumab govitecan

Sacituzumab govitecan (SG) consists of a fully humanized IgG1 anti-Trop-2 antibody conjugated to the payload SN-38, a topoisomerase I inhibitor. This ADC is characterized by a high drug-to-antibody ratio (7.5–8), allowed by the significantly better toxicity profile of SN-38 than irinotecan, its prodrug. The mAb is linked to SN-38 by a hydrolysable linker, which causes the release of some drug molecules into the tumor microenvironment, leading to killing nearby tumor cells via the bystander effect [49].

In April 2020, SG was granted accelerated FDA approval as a treatment for triple-negative breast cancer after at least two prior therapies for metastatic disease, and has recently received fast-track designation for NSCLC and urothelial carcinoma.

SG was firstly investigated in the phase I/II, single arm IMMU-132-01 study (NCT01631552), in which 108 patients with TNBC pretreated with at least two regimens for metastatic disease were enrolled. Impressive responses were seen in terms of both ORR (33.3%) and DOR (median of 7.7 months), the two primary study endpoints. Notably, the median duration of treatment with SG (5.1 months) was approximately twice that with the previous anticancer therapy (2.5 months). Most frequently observed AEs of any-grade were nausea, neutropenia, diarrhea, fatigue and anemia. Neutropenia, anemia and leucopenia were also the most common AEs of grade 3 or higher (> 5% incidence). 7% of patients had febrile neutropenia [50].

Data from the randomized, phase III, ASCENT trial have been recently presented at 2020 ESMO Congress. SG outperformed standard chemotherapy (capecitabine, eribulin, vinorelbine or gemcitabine as per physician’s choice) in terms of both PFS (median PFS of 5.6 vs 1.7 months) and OS (median OS 12.1 vs 6.7 months), with a safety profile similar to what observed in earlier trials [51].

As for nectin-4, also Trop-2 expression was not requested to select patients for SG therapy. An earlier analysis from the IMMU-132–01 trial performed on 48 archival samples showed a moderate (2+) to strong (3+) IHC expression in 88% of patients. Among patients evaluable for response, all responders had 2+ or 3+ staining (16 responses over 46 evaluable patients), whereas patients with weak (1+) to no expression (0) achieved disease stability as best response [52]. No data are available about efficacy of SG in patients stratified for Trop2 expression in patients enrolled in the ASCENT trial.

##### HR+/HER2− BC

Trop-2 is frequently expressed not only in TN subtype, but also in hormone-receptor (HR)-positive BC. SG activity has been consequently explored also in this BC subtype and promising results recently emerged from the HR+/HER2−mBC cohort of the IMMU-132-01 basket trial. At a median follow-up of 11.5 months, the ORR was 31.5%, median DOR was 8.7 months, median PFS was 5.5 months, and median OS was 12 months [53]. Even if preliminary, responses and clinical activity here achieved are significant when compared to the alternative standard regimens for heavily pretreated HR+ mBC, in which chemotherapy provides poor benefit. Further evaluation in a randomized phase III trial (TROPiCS-02) is ongoing (NCT03901339).

##### Urothelial carcinoma

The urothelial cancer cohort of the same basket trial also provided encouraging data, with an observed ORR of 31% and a median PFS of 7.3 months [54]. The phase II TROPHY U-01 trial (NCT03547973) confirmed these data in patients with mUC progressing on both platinum-based chemotherapy and immune-checkpoint inhibitors (cohort 1), reporting an ORR of 27%, a median DOR of 5.9 months and median PFS and OS of 5.4 months and 10.5 months, respectively [55]. A confirmatory phase III trial (TROPiCS-04 Trial; NCT04527991) is enrolling patients in the same setting, while cohort 3 of the TROPHY U-01 trial recently opened to immunotherapy-naïve patients progressing to a platinum-based chemotherapy.

##### Selecting patients for Trop-2 expression: the TROPiCS-03 study

The abovementioned phase 1/2 IMMU-132-01 basket study showed antitumor activity in patients with tumor types other than mBC and mUC, including NSCLC (ORR 17%) or metastatic endometrial carcinoma (mEC) (ORR 22.2%) [56, 57]. None of these trials selected patients for Trop-2 expression.

In order to investigate if a different strategy might increase efficacy of SG, a different biomarker-oriented basket trial was designed. The TROPiCS-03 (NCT03964727) is a multi-cohort, open-label, phase 2 study that is currently open to enrollment for patients with metastatic solid tumors, presently NSCLC (adenocarcinoma and squamous cell carcinoma), head and neck squamous cell carcinoma, and endometrial cancer, all selected for Trop-2 overexpression by a validated IHC assay.

## Promising new targets under investigation

### LIV1

The LIV-1 family identifies a subgroup of transmembrane proteins belonging to the ZIP superfamily of zinc transporter [58]. Their expression in normal tissues is heterogeneous, reflecting their specific role in different cell types. Among them, LIV1 (ZIP6) protein is mainly detected in hormonally controlled tissues, including breast, where its expression was found to be sensitive to estrogen levels [59]. In cancer cells it has been firstly identified as an estrogen-induced gene in breast cancer cell lines [60] and subsequently associated to node involvement in HR-positive breast cancer [59]. In addition to breast cancer, it has been detected in pancreatic, prostate, melanoma, cervical and uterine carcinomas [58, 61].

#### Ladiratuzumab vedotin (SGN-LIV1A)

Ladiratuzumab vedotin (LV) is an anti-LIV1 ADCs carrying the MMAE payload via a protease-cleavable linker. The SGNLVA-001 phase I study firstly investigated increasing doses of LV in 69 patients with mBC (8HR+/HER2−, 51 TN) [62]. Treatment was overall well tolerated, with a toxicity profile similar to what observed with other MMAE-based ADCs. Fatigue (59%), nausea (51%), peripheral neuropathy (44%), alopecia (36%), decreased appetite (33%), constipation (30%), abdominal pain (25%), diarrhea (25%) and neutropenia (25%) were the most frequent all-grade AEs. Neutropenia (25%) and anemia (15%) were reported as grade ≥ 3 AEs. Preliminary antitumor activity was observed in both HR+/HER2−(DCR 59%) and TNBC (DCR 64%) subgroups. Of note, 631 tumor samples were evaluated for LIV1 expression (all-subtypes considered), with a 91% rate of LIV1-expression and a 75% of cases with moderate-to-high expression.

Antitumor activity of LV is primarily mediated by apoptotic death. Nevertheless, preclinical evidence in TNBC cell lines showed also that LV is capable of inducing an effective immunogenic cell death (ICD), so ideally increasing the potential benefit of immunotherapy [63]. The combination of pembrolizumab and LV is currently under investigation as first-line therapy for mTNBC in the phase I/II SGNLVA-002 [64], while one arm of the Morpheus-TNBC trial is evaluating the administration of atezolizumab plus LV as second-line treatment. Finally, LV is under investigation in the phase II SGNLVA-005 study [65], enrolling patient with advanced solid tumors in different tumor-specific cohorts (GC and GEJ adenocarcinoma, esophageal squamous cell carcinoma, SCLC, squamous-NSCLC, non-squamous-NSCLC and head and neck squamous cell carcinoma). Ongoing trials investigating LV are reported in Table 2.

**Table 2:** Selected novel ADCs under evaluation (completed/withdrawed trials are not reported); *basket trial

Antibody–drug conjugate	Patload	Trial	Phase	Population	Treatment arm(S)	Biomarker
*CEACAM5*						
SAR408701	DM4 (tubulin inhibitor)	NCT02187848	I/II	Advanced solid tumors	SAR408701 monotherapy	No
		NCT04524689 (CARMEN-LC05)	II	Non-squamous mNSCLC (wild type for EGFR, ALK/ROS1, BRAF)	SAR408701 + pembrolizumab; pembrolizumab monotherapy	Yes
		NCT04394624 (CARMEN-LC04)	II	Non-squamous mNSCLC	SAR408701 + ramucirumab	yes
		NCT04154956 (CARMEN-LC04)	III	Non-squamous mNSCLC	SAR408701 monotherapy; docetaxel	Yes
*c-MET*						
Telisotuzumab vedotin (teliso-V)	MMAE (tubulin inhibitor)	NCT02099058	I	Advanced solid tumors (mNSCLC in dose expansion)	Teliso-V monotherapy Teliso-V + erlotinib (NSCLC) Teliso-V + nivolumab (NSCLC) Teliso-V + osimertinib (NSCLC)	Yes (dose expansion)
		NCT03539536	II	mNSCLC	Telisotuzumab vedotin monotherapy	Yes
TR1801-ADC	Pyrrolobenzodiazepine (DNA-crosslinking agent)	NCT03859752	I	Advanced solid tumors	TR1801-ADC monotherapy	Yes
SHR-A1403	Novel microtubule inhibitor	NCT03856541	I	Advanced solid tumors	SHR-A1403 monotherapy	No
*FOLATE RECEPTOR ALPHA*						
Mirvetuximab soravtansine (IMGN853)	DM4 (tubulin inhibitor)	NCT02606305	I/II	Advanced OC, fallopian tube, primary peritoneal cancer	IMGN853 + bevacizumab; IMGN853 + carboplatin; IMGN853 + pegylated liposomal doxorubicin; IMGN853 + pembrolizumab; IMGN853 + bevacizumab + carboplatin	Yes
		NCT03832361	II	mEC	IMGN853 monotherapy	Yes
		NCT02996825	I	Recurrent OC, primary peritoneal, fallopian tube, mEC, mTNBC	IMGN853 + gemcitabine	Yes
		NCT03552471	I	Advanced OC, fallopian tube, primary peritoneal cancer or mEC	IMGN853 + rucaparib	Yes
		NCT03835819	II	mEC	IMGN853 + pembrolizumab	Yes
		NCT04606914	II	Advance-stage OC, fallopian tube, primary peritoneal cancer (neoadjuvant setting)	IMGN853 + carboplatin	Yes
		NCT04274426 (MIROVA)	II	Recurrent OC, fallopian tube, primary peritoneal cancer	IMGN853 + carboplatin; Platinum-based chemotherapy	Yes
		NCT04209855 (MIRASOL)	III	Advanced OC, fallopian tube, primary peritoneal cancer	IMGN853; Chemotherapy of investigator’s choice (paclitaxel or topotecan or pegylated liposomal doxorubicin)	Yes
		NCT04296890 (SORAYA)	III	mOC, fallopian tube, primary peritoneal cancer	IMGN853 monotherapy	Yes
STRO-002	Hemiasterlin (tubulin inhibitor)	NCT03748186	I	Advanced OC, fallopian tube, primary peritoneal cancer, mEC	STRO-002 monotherapy	No
MORAb-202	Eribulin (tubulin inhibitor)	NCT03386942	I	Advanced solid tumors	MORAb-202 monotherapy	Yes (only for selected subtypes)
		NCT04300556	I/II	mOC, fallopian tube, primary peritoneal cancer, mTNBC, mEC, NSCLC adenocarcinoma	MORAb-202 monotherapy	Yes (dose expansion)
*HER3*						
Patritumab deruxtecan (U3-1402)	Deruxtecan (TOP1 inhibitor)	NCT02980341	I/II	mBC	Patritumab deruxtecan monotherapy	Yes
		NCT03260491	I	mNSCLC	Patritumab deruxtecan monotherapy	No
		NCT04479436	II	mCRC	Patritumab deruxtecan monotherapy	2 cohorts (IHC 2+/3+; 1+/0)
		NCT04610528	Early phase I	HR+/HER2- eBC (treatment-naïve patients)	Patritumab deruxtecan monotherapy	4 cohorts
		NCT04619004 (HERTHENA-Lung01)	II	EGFR-mutated mNSCLC	Patritumab deruxtecan monotherapy	No
*LIV-1*						
Ladiratuzumab vedotin (SGN-LIV1A)	MMAE (tubulin inhibitor)	NCT01969643	I	mBC	SGN-LIV1A monotherapy; SGN-LIV1A + trastuzumab (part B)	No
		NCT03310957	Ib/II	mTNBC (first-line setting)	SGN-LIV1A + pembrolizumab	No
		NCT04032704	II	Advanced SCLC, NSCLC, HNSCC, ESCC, GC, GEJC, prostate cancer, melanoma	SGN-LIV1A monotherapy	No
		NCT03424005 (Morpheus-TNBC)*	I/II	mTNBC	SGN-LIV1A + atezolizumab	No
		NCT01042379 (I-SPY2)*	I	Triple-negative eBC (neoadjuvant setting)	SGN-LIV1A monotherapy	No
*MESOTHELIN*						
Anetumab ravtansine	DM4 (tubulin inhibitor)	NCT03126630	I/II	MPM	Anetumab ravtansine + pembrolizumab; Pembrolizumab monotherapy	Yes
		NCT03102320	I	Advanced solid tumors	Anetumab ravtansine monotherapy; Anetumab ravtansine + gemcitabine; Anetumab ravtansine + cisplatin	Yes
		NCT03587311	I/II	Advanced OC, fallopian tube, or primary peritoneal cancer	Anetumab ravtansine + bevacizumab; Paclitaxel + bevacizumab	Yes (only for part 2)
		NCT03816358	I/II	Pancreatic adenocarcinoma	Anetumab ravtansine + nivolumab; Anetumab ravtansine + nivolumab + ipilimumab; Anetumab ravtansine + nivolumab + gemcitabine	Yes
RC88	MMAE (tubulin inhibitor)	NCT04175847	I	Advanced solid tumors	RC88 monotherapy	Yes
BMS-986148	Tubulysin (tubulin inhibitor)	NCT02341625	I/II	MPM, NSCLC, OC, GC, pancreatic cancer	BMS-986148 monotherapy; BMS-986148 + nivolumab	Yes (dose expansion)
*TISSUE FACTOR*						
Tisotumab vedotin	MMAE (tubulin inhibitor)	NCT03485209 (InnovaTV 207)	II	mCRC, pancreatic cancer, squamous NSCLC, HNSCC	Tisotumab vedotin monotherapy	No
		NCT03438396	II	mCC	Tisotumab vedotin monotherapy	No
		NCT03657043 (InnovaTV 208)	II	Advanced OC, fallopian tube, peritoneal cancer	Tisotumab vedotin monotherapy	No
		NCT03786081	I/II	mCC	Tisotumab vedotin monotherapy Tisotumab vedotin + carboplatin Tisotumab vedotin + pembrolizumab Tisotumab vedotin + bevacizumab	No

### CEACAM

The carcinoembryonic antigen-related cell adhesion molecules (CEACAMs) are a family of 12 immunoglobulin-related proteins physiologically expressed on cell membranes of many epithelial tissues, where they act as modulators of different processes such as cell adhesion, differentiation, proliferation and survival [66]. The link between CEACAMs and cancer has been established several years ago with the identification of CEA (CEACAM5) as tumor biomarker, and later with the description of CEACAMs’ role as signal modulators involved in cancer progression and metastasis [67]. Their role as therapeutic target has been further explored, mainly throughout investigational agents targeting CEACAM1, CEACAM5 or CEACAM6, the ones best characterized in cancer processes. In particular, CEACAM5 is highly expressed by several tumor types, including CRC, lung and gastric tumors, whereas its expression in normal tissue is limited to columnar absorptive cells [68].

#### SAR408701

SAR408701 is an ADC in which an anti-CEACAM5 antibody is conjugated to DM4, a maytansinoid cytotoxic agent. In the first-in-human study [69], 31 pts were treated across 8 dose levels (from 5 to 150 mg/m^2^), including CRC (18), GC (7), GEJ (3), esophagus (1), BC (1) and pancreas (PC) (1) cancers. CEACAM5 expression was assessed retrospectively by IHC. Most frequent AEs (≥ 20%) were fatigue/asthenia (32%), nausea, neuropathies and decreased appetite (26%), diarrhea, constipation and keratopathy (23%). Dose-limiting toxicities (DLTs) occurred in 5 patients due to reversible G3 keratopathy, and the maximum-tolerated dose (MTD) was defined at 100 mg/m^2^ Q2W. Given the manageable tolerability profile, SAR408701 was then subsequently investigated in three expansion cohorts, enrolling patients with CRC, lung cancers (both small-cell lung cancer and non-squamous NSCLC) and GC. Most promising data came from the non-squamous NSCLC cohort [70], where a high antitumor activity was shown particularly in patients with high expression of CEACAM5 (≥ 50%; ORR 20.3%, SD 42.2%), while responses were reduced in moderate expressors (1–49%; ORR 7.1%). Accordingly, subsequent development of SAR408701 was mainly focused on patients with CEACAM5-positive non-squamous NSCLC, and three ongoing trials are investigating it as monotherapy (CARMEN-LC03), in association with ramucirumab (CARMEN-LC04) or as first-line therapy in combination with PD-1 (CARMEN-LC05) are ongoing (Table 2).

Of note, despite the high expression of CEACAM5 by colorectal cancer cells, development of SAR408701 was discontinued in this subtype because of its low sensitivity to microtubule-inhibitors payload.

#### Labetuzumab govitecan (IMMU-130)

Labetuzumab govitecan (LG) is another anti-CEACAM5 ADC in which the antibody labetuzumab is conjugated to SN-38 by a pH-sensitive linker. The payload is the active metabolite of irinotecan, the same carried by SG, and as the latter, a high DAR (7.8) characterizes this ADC. Two phase I studies (IMMU-130-01; IMMU-103-02) [71] initially tested LG in patients with mCRC after standard treatments, including irinotecan, and having elevated plasma CEA. Neutropenia and diarrhea were the major side effects, but manageable, and initial therapeutic activity was observed. A subsequent phase I/II trial [72] investigated different dose regimens and identified the once weekly regimen as the preferred one. In this study, LG did not show major responses (except one long-lasting PR), and a clinical benefit rate of 29% (25 of 86) was observed. In the overall population, median PFS was 3.6 months, and median OS was 6.9 months. Considering the activity of LG in patients progressing on irinotecan-based regimens and the relatively low efficacy of currently available third-line regimens in mCRC, authors postulated a possible role of LG in this setting, or alternatively in earlier lines in combination with other agents. Nevertheless, currently there are no studies investigating this agent.

### HER3

HER3 (ErbB3) is a member of the HER family with very faint tyrosine kinase activity, that has to form heterodimers in order to fully transduce downstream signals, with HER2 being the most important [73]. Neuregulins (NRG-1 and NRG-2) are well-characterized high affinity ligands of HER3 [74].

Overexpression of HER3 is common in a wide variety of human cancer, including breast, ovarian, prostate, lung, melanoma, bladder, colorectal and head and neck squamous cell carcinoma [75]. Several studies demonstrated a putative role of HER3 in mediating resistance to targeted therapies against other receptors of the HER family (mainly HER2), PI3K-inhibitors or hormonal agents. Apart from overexpression, ERBB3 somatic mutations also showed oncogenic potential [76].

#### Patritumab deruxtecan (U3-1402)

Patritumab deruxtecan (HER3-DXd) is a novel HER3-targeted ADC that conjugate the mAb patritumab with deruxtecan, via a peptide-based cleavable linker. As other deruxtecan-based ADCs, high DAR (8) and membrane permeability, thus able to elicit a potent bystander effect, characterize it. This first-in-class agent has been firstly explored in a phase I/II study enrolling patients with HER3-positive breast cancer, with impressive results [77]. The ORR and DCR achieved were 42.9% and 90.5%, respectively, in a population of heavily pretreated HER3-expressing MBC patients (median prior anticancer regimens of 6). Most frequent all-grade AEs were gastrointestinal (nausea 85.7%, vomiting 54.8%) and hematological toxicities (thrombocytopenia 71.4%, neutropenia 64.3%, leukopenia 59.5%, and anemia 38.1%), along with appetite reduction (66.7%) and AST/ALT increasing (47.6% and 45.2%, respectively). Thrombocytopenia (35.7%), neutropenia (28.6%), leukopenia (21.4%) and anemia (16.7%) were also the most common grade ≥ 3 AEs [77].

In NSCLC, HER3-overexpression has been reported in 80% of EGFR-mutated adenocarcinomas [78]. HER3-DXd demonstrated to inhibit tumor growth in HER3+, EGFR-mutated, EGFR-TKI resistant patient-derived xenograft (PDX) models, while it was ineffective in HER3-negative models [79]. A phase I trial then investigated this ADC in EGFR-mutated NSCLC progressing to first generation EGFR-TKIs and negative for T790M mutation, or after osimertinib failure [80]. In the dose-expansion phase, all patients must have received also a platinum-based chemotherapy. The reported safety profile was similar to what observed for BC, with hematological toxicities, nausea and fatigue as most frequent AEs. Antitumor activity was seen regardless of the specific mechanism of resistance to EGFR-TKI (EGFR mutations, amplifications of non-EGFR mutations/fusions). The observed ORR and DCR were 25% and 70%, respectively, with a median DOR of 7 months. Of note, patients were unselected for HER3-expression. The ongoing phase II HERTHENA-Lung01 study is currently evaluating patritumab deruxtecan in patients with metastatic NSCLC progressed on or after at least one EGFR-TKI and one platinum-based chemotherapy.

HER3-overexpression is common also in CRC, where it has been detected in 20–75% of cases [81]. A phase II study evaluating patritumab deruxtecan in patients with advanced/metastatic CRC who are resistant, refractory, or intolerant to at least two previous lines of systemic therapy is ongoing (NCT04479436). Table 2 reports ongoing trials investigating HER3-DXd across several tumor types.

### Mesothelin

Mesothelin is a membrane protein normally expressed by mesothelial cells lining the pleura, pericardium and peritoneum, whose biological function is unknown. Its overexpression has been found in several human malignancies, including mesothelioma, ovarian cancer, NSCLC, pancreatic adenocarcinoma, breast cancer, gastric cancer and cholangiocarcinoma. The contribution of mesothelin in cancer development and progression is barely understood, with a clearly defined role only in metastatic spread of ovarian cancer [82]. Nevertheless, the high expression of mesothelin by cancer cells and its low expression on normal tissue made this protein an ideal tumor-associated antigen, and led to development of several anti-mesothelin targeted agents [83], including mAbs, vaccines, immunotoxins and lastly antibody–drug conjugates.

#### Anetumab ravtansine (BAY 94-9343)

Anetumab ravtansine is an ADC comprising a fully humanized IgG1 anti-mesothelin mAb conjugated to the tubulin inhibitor DM4 by a reducible disulfide linker. Preclinical evidence showed promising activity in both cell lines and PDX from different tumor types, including mesothelioma, pancreatic cancer, ovarian carcinoma and NSCLC [84]. The first-in-human study evaluated anetumab ravtansine in patients with advanced solid tumors, enriched for cancer with known mesothelin overexpression (mesothelioma, ovarian and peritoneal cancer) [85]. Expression of mesothelin was evaluated by IHC, retrospectively in dose-escalation and once-every-3-weeks expansion cohorts, and prospectively in the once-weekly dose-expansion cohorts that included only tumors with high mesothelin expression (2+ or 3+). Most frequently observed drug-related AEs at the defined RP2D of 6.5 mg/kg were fatigue, nausea, diarrhea, anorexia, vomiting and peripheral sensory neuropathy, mainly reported as grade 1–2. Most frequent ≥ 3 drug-related AEs were fatigue, keratitis/keratopathy and nausea. In the overall population evaluable for antitumor response (138 patients), 48% of patients had SD, 8% had a PR, and 1 CR was observed. The highest activity was seen among patients with mesothelioma treated with anetumab ravtansine at 6.5 mg/kg every 3 weeks (ORR 31%; DCR 75%), while few responses were observed in patients with ovarian cancer. Notably, all responding patients had at least 60% tumor mesothelin expression (2+ or 3+ by IHC), with 14 patients on treatment for more than 200 days (9 mesothelioma and 5 ovarian cancers).

Strengthened by these findings, anetumab ravtansine was investigated versus vinorelbine as second-line treatment for advanced mesothelioma with high-mesothelin expression, after failure of first-line platinum/pemetrexed chemotherapy. Unexpectedly, this phase II trial failed to demonstrate the superiority of anetumab ravtansine, with a median PFS of 4.3 versus 4.5 months for vinorelbine [86]. Many other trials are underway in several tumor types, investigating this agent alone or in combination with chemotherapy, anti-angiogenic agents or immune-checkpoint inhibitors (ICIs). Of note, two trials enrolling patients with NSCLC closed prematurely because of slow accrual.

Others anti-mesothelin ADCs, like DMOT4039A, RC88 and BMS-986148, are under clinical evaluation (Table 2). The latter showed promising results when combined with nivolumab in patients with mesothelin-selected mesothelioma [87].

### c-Met

c-Met, also known as hepatocyte growth factor receptor (HGFR), is a membrane receptor with tyrosine kinase activity. Upon binding with HGF, its ligand c-Met activates several different signaling pathways, including PI3K/AKT, Ras/MAPK, JAK/STAT, SRC and Wnt/β-catenin [88]. Physiologically, c-Met is primarily implicated in processes of cell motility occurring during embryogenesis and wound repair, while its pathological activation throughout mutation, amplification or overexpression has been detected in different cancer types. Aberrations of c-Met have been described as both primary events, leading to cancer cell transformation, and secondary events, responsible for cancer progression and treatment resistance [89]. Targeting c-Met has been widely explored throughout both mAbs and small tyrosine-kinase inhibitors (TKIs), which demonstrated to be active against tumors with MET amplification or MET exon 14 mutations, but less in tumors overexpressing c-Met through other mechanisms [88]. Anti-cMet ADCs have been then developed with the goal of expanding the population sensitive to c-Met targeting, by addressing also tumors not strictly addicted to the c-Met pathway.

#### Telisotuzumab vedotin

Telisotuzumab vedotin (Teliso-V) is a first-in-class ADC that couples the anti-c-Met humanized mAb with MMAE through a peptide linker. The first-in-human phase I study enrolled 48 patients with pretreated (3 median number of prior lines) advanced solid tumors, 39 unselected for c-Met expression in the dose-escalation cohort and 9 patients with c-Met-positive NSCLC in the dose-expansion cohort [90]. c-Met expression was assessed by IHC, and an H-score cutoff of ≥ 150 was chosen to define c-Met positivity based on preclinical data. c-Met status was assessed retrospectively for patients in the dose-escalation cohort and prospectively for patients in the dose-expansion phase. Of note, 60% of patients with NSCLC screened in this trial was identified as c-Met positive. Most frequent all-grade treatment-related AEs were fatigue (25%), gastro-intestinal toxicity (nausea 23%, vomiting 13%, and diarrhea 10%), and neuropathy (15%). Few high-grade (≥ 3) events were ascribed to teliso-V (fatigue, hypoalbuminemia, anemia and neutropenia, all of them in 2 patients). In the overall population, twenty-five patients (52.1%) experienced disease control as best response, mainly as disease stability (22 SD, 3 PR, 0 CR). Of note, all responding patients had squamous-NSCLC, and 2 patients with lung adenocarcinoma had a significant tumor shrinkage even if not reaching the cut-off for RECIST partial response [90].

Hence, subsequent development of TV focused on NSCLC. The phase Ib of the abovementioned study included only patients with NSCLC and investigated teliso-V in combination with erlotinib (arm A), nivolumab (arm D), or osimertinib (arm E). Among the 29 patients with c-Met-positive, EGFR-mutated NSCLC treated in arm A and evaluable for response, an ORR of 34.5% was observed, with a DCR of 86.2%. Noteworthy, all patients were pretreated with an anti-EGFR TKI, and 69% of them received ≥ 3 prior lines of therapy [91]. Arm E is enrolling patients with EGFR-mutated NSCLC after progression to osimertinib, including only patients whit c-Met overexpression assessed on tumor tissue obtained post-osimertinib progression [92]. Conversely, data from LUNG-MAP sub-study [93] S1400K evaluating teliso-V in squamous NSCLC in ICI-refractory or ICI-naïve patients were disappointing, with a ORR of 9% (2/23) and 7 high-grade events (3 G5 and 4 G3), leading to S1400K closure.

A phase II study (NCT03539536) investigating teliso-V in both squamous and non-squamous (EGFR mutated and wt) c-Met-positive NSCLC is open to enrollment, with data expected in 2021.

Apart from teliso-V, two others anti-cMet ADCs (TR1801-ADC and SHR-A1403) are currently under investigation in advancer solid tumors, with promising preclinical activity [94, 95] (Table 2).

### Folate receptor alpha

Folate receptor alpha (FRα) is glycosyl-phosphatidylinositol (GPI)-anchored membrane glycoprotein barely expressed by several normal tissues, including the choroid plexus, thyroid and salivary glands, breast, colon and bladder [96, 97]. Despite having a recognized crucial role in embryogenesis, the function of FRα in adult tissue is less understood. This receptor has been found to be overexpressed across several cancer types, including mesothelioma (72–100%), ovarian cancer (76–89%), TNBC (35–68%), NSCLC (14–74%) and endometrial cancer (20–50%) [97, 98], where it seems to be involved in regulation of cell motility, invasion and proliferation. Given its limited expression on normal cells, its putative role in cancer progression, and a proved high-affinity for non-natural ligands like folic acid, FRα is an attractive therapeutic target for different biological drugs, including mAbs, small-molecule inhibitors ADCs, bispecific antibodies and CAR-T cells.

#### Mirvetuximab soravtansine

Mirvetuximab soravtansine (MS) is an ADC in which a humanized anti-FRα mAb is conjugated with the antimitotic agent DM4 through a cleavable linker. Upon promising antitumor activity on preclinical models, it was firstly evaluated in a phase I trial on forty-four patients affected by advanced solid tumors with high probability of FRα expression (epithelial ovarian cancer (EOC), primary peritoneal cancer, fallopian tube cancer, serous or endometrioid endometrial cancer, non-squamous NSCLC, and renal cell cancer) [99]. After dose-escalation, the recommended phase-2 dose was assessed at 6 mg/kg based on adjusted ideal body weight (AIBW) every 3 weeks. Treatment was overall well tolerated, with only 6 reported grade 3 AEs, each of them in one patient only. Most common all grade, treatment-related AEs were fatigue (25%), blurred vision (22.7%), diarrhea (34%), peripheral neuropathy (20.5%), ALT increased (15.9%) and keratopathy (15.9%). Ten over forty-three (23%) patients evaluable for tumor response achieved a clinical benefit (ORR + SD ≥ 4 months + CA 125 response), mostly observed in patients with EOC (7 out of 10). Of them, two patients had a partial response. These promising data in patients with EOC led to opening different expansion cohorts for this cancer subtype, which confirmed the high ORR (22% and 26% in two different cohorts [99, 100] and 47% in a pooled analysis [101]) and allowed a better understanding of the relationship between FRα expression and response. Indeed, a correlation between higher FRα expression by IHC and greater antitumor activity was observed, and a good level of concordance (71%) among FRα levels in archival tissues and fresh biopsies was demonstrated, despite treatments and interval time elapsed between the two samplings.

Mirvetuximab soravtansine was then evaluated in the pivotal phase III FORWARD I study, investigating MS monotherapy versus TPC in patients with platinum-resistant EOC, fallopian tube or peritoneal cancer pretreated with 1–3 lines of therapy. Only tumors with medium/high FRα could have been enrolled (≥ 50% of cells with ≥ 2+ staining). According to preliminary data, MS failed to show an advantage over chemotherapy in the ITT population, with a median PFS of 4.1 versus 4.4 months, respectively. Slightly better results were achieved in the high FRα subpopulation, with a median PFS of 4.8 versus 3.3 months (still not significant) and an ORR of 24% (vs 10% in the standard arm) [102]. OS data are maturing. Data from the MIRASOL phase III trial [103], which is investigating MS versus TPC in a similar population but restricted to patients with high FRα expressing tumors (≥ 75% of cells with PS2+ staining intensity), are awaited.

Alternative strategies combining mirvetuximab soravtansine with other anticancer agents in patients with EOC are also underway. The phase Ib/II FORWARD II study [104] includes five possible associations (bevacizumab, carboplatin, pegylated liposomal doxorubicin, pembrolizumab or bevacizumab + carboplatin), while another trial is evaluating MS in combination with rucaparib (NCT03552471). Results from these combinations showed a good tolerability profile along with promising activity, thus justifying a subsequent development in larger trials. Of note, a phase II study investigating mirvetuximab soravtansine in FRα-expressing TNBC was closed prematurely because of a low rate of FRα positivity in the screened population and lack of response in two patients treated [105]. Other studies enrolling patients with endometrial cancers are underway (Table 2).

### Tissue factor

Tissue factor (TF) is a transmembrane glycoprotein normally expressed by sub-endothelial cells and fibroblasts, with an essential role in hemostasis regulation [106]. Indeed, the binding between factor VII (FVII) and TF generates the active TF:FVIIa complex responsible for activation of the coagulation extrinsic cascade. Physiologically, this pathway is induced by vascular injury, which lead to exposure of normally hidden TF-positive cells to blood [106]. Conversely, TF is frequently expressed by tumor cells and showed to promote tumor growth, angiogenesis, metastasis and thrombosis [107]. Because of its selective expression limited on normal cells and increased on malignant cells, TF is currently explored as potential target for mAbs, small TKIs and ADCs.

#### Tisotumab vedotin

Tisotumab vedotin (TV) is a first-in-human ADC in which a fully humanized anti-TF mAb (HuMax-TF) is conjugated with the MMAE payload, through a protease-cleavable valine citrulline linker. Preclinical evidence about TV or HuMax-TF alone showed that differently from other TF-targeting mAb, it does not alter coagulation parameters while maintaining high cytotoxic power [108]. InnovaTV 201 [109] is the first-in-human trial that investigated tisotumab vedotin in tumors known to express TF and sensitive to microtubule-inhibitors agents, including patients with ovarian, cervical, esophageal, bladder, endometrial, prostate, head and neck SCC (HNSCC) and NSCLC. Most common AEs of any grade were epistaxis (69%), fatigue (56%), nausea (52%), alopecia (44%), conjunctivitis (43%), decreased appetite (36%), constipation (35%), diarrhea (30%), vomiting (29%), peripheral neuropathy (22%), dry eye (22%) and abdominal pain (20%). Toxicity profile was consistent with what observed for other MMAE-based ADCs, except for epistaxis, which was related to TF targeting. Of note, all cases of epistaxis were low grade (98% grade 1). Few high grade AEs were reported, of which the most frequent were fatigue (10%), anemia (5%), abdominal pain (4%) and hypokalemia (4%). Some RECIST responses were recorded across several tumor types, with an ORR of 15.6% and remarkable activity in cervical cancer [110]. Accordingly, the phase II InnovaTV 204 [111] tested TV exclusively in patients with pretreated recurrent or metastatic CC (r/mCC) progressing to chemotherapy plus bevacizumab. Activity of TV was confirmed, with an ORR of 24%, a DCR of 72% and a median DOR of 8.3 months. Median PFS and OS were 4.2 and 12.1 months, respectively. Safety profile was similar to the one reported above, even if more high grade, treatment-related AEs of interest were reported (grade 3 ocular, bleeding, and peripheral neuropathy in 2%, 2%, and 7% of patients, respectively). A phase Ib/II trial investigating TV monotherapy or in combination with bevacizumab, pembrolizumab, or carboplatin in patients with untreated or previously treated r/mCC is ongoing (InnovaTV 205) [112]. Other ongoing trials evaluating TV across several tumor types are reported in Table 2.

## Discussion

Three new ADCs have been recently added to the therapeutic armamentarium of solid cancers, and many others are under development with promising preliminary data.

Sacituzumab govitecan and enfortumab vedotin firstly included Trop-2 and nectin-4 in the landscape of druggable tumor targets. Meanwhile, trastuzumab deruxtecan significantly broadened targetability of HER2, both in terms of tumor types and therapeutic index. Main reasons behind the success of these agents lie in biochemical improvements in ADC development. Indeed, the timely progress in technology platforms and ADCs engineering has introduced novel linkers, new payloads and allowed the creation of new generation of ADCs with high DAR and potent bystander effects. For instance, discovery of new warheads with mechanisms of action different from tubulin-inhibitions allowed expanding ADCs applicability to tumor types usually unresponsive to anti-microtubule agents. This was made possible primarily by the capability of charging mAb with a high number of payload molecules, i.e., increasing the DAR, so that less cytotoxic drugs could also be exploited as payloads given the high intracellular concentration. Furthermore, the use of new cleavable linkers along with membrane-permeable payload maximized the efficacy of bystander effect, making ADCs active also against target-negative cells, hence extending efficacy to heterogeneous tumors or cancers with homogeneous but low target expression.

In order to further increase the population of patients that might benefit from these new generation drugs, several new strategies have been conceived.

Firstly, new potential targets other than tumor-associated antigens are under evaluation, as proteins expressed in the tumor microenvironment (TME) or by cancer stem cells (CSC). CD25, CD205, B7-H3 are targets found in the TME for which specific ADCs reached the clinical phases of drug development, as delta-like ligand 3 (DLL3), ﻿ephrin-A4, ﻿PTK7 and 5T4 among CSC-associated [4].

Other lines of development are focusing their attention on smarter vehicles for payloads. Probody drug conjugates are a new class of recombinant, ADCs prodrugs that circulate in an inactivated form, to be activated after a proteolytic cleavage by TME proteases. This expedient minimizes on-target/off-tumor toxicity, by delivering the payload only at the tumor site. Good safety profile along with preliminary tumor activity has been observed in first-in-human studies testing this new class of ADCs [113, 114]. Alternative delivery systems that do not use antibody scaffolds have also been explored. Centyrins are small (∼ 10 kDa) cysteine-free scaffolds based on the type-III fibronectin domain of human Tenascin C. These alternative scaffolds possess excellent biophysical properties, which allow for mutation and conjugation at one or even multiple positions and are easily internalized by tumor [115]. Biparatopic and bispecific antibodies are also under evaluation in preclinical models. The first binds two non-overlapping epitopes of the same antigen, while the second can recognize two different antigens on the same antigen [116, 117].

Moreover, payload alternative to cytotoxic agents is under development. LMB-100 and ABBV-155 are two experimental ADCs that incorporate proapoptotic payloads, the first the pseudomonas exotoxin A, the latter a Bcl-2 inhibitors. Similarly, other ADCs deliver immunomodulatory agents, such as TLR7/8 [4].

In order to further increase efficacy of novel ADCs, several ongoing trials are testing these agents in combination with other drugs as targeted agents, chemotherapy, or ICIs (Table 2). Of note, many of the abovementioned ADCs showed to induce ICD, thus potentially boosting immunotherapy activity [118, 119].

Despite continuous progresses in the field of ADCs, some challenges still need to be tackled. Safety profiles of novel ADCs are usually favorable, even if some disabling or potentially fatal toxicities have been described. These AEs can be caused by both on-target/off-tumor toxicities and/or by side effects of the payload itself. Trastuzumab deruxtecan has been associated with development of grade 5 interstitial lung diseases (ILDs) across several tumor types, while maytansinoids or auristatins-based ADCs have been linked to high-grade ocular or neurological toxicities, not always reversible. Noteworthy, rovalpituzumab tesirine and PSMA-ADC, two promising ADCs, respectively, targeting DLL3 and the prostate specific membrane antigens (PSMA) were both recently discontinued. They were characterized by not easily manageable toxicities, which might have contributed to the failure of these agents. Consequently, toxicity profile of ADCs should be always taken into account when selecting patients, all prophylactic measures should be implemented, and a high level of attention must be kept toward these rare but potentially fatal events. Optimal selection of patients for clinical trials evaluating ADCs is another open question. As abovementioned, some trials are adopting a prescreening phase to enroll only patients expressing the specific target, while others limit inclusion to tumor types known to have a higher target expression without testing the individual patient. Both these strategies can be inconvenient, the first because may lead negative patients to wasting time, the second because may expose unresponsive patients to unnecessary toxicity. On the other hand, ADCs like T-DXd showed to induce responses also in patients with reduced target expression. This issue is even more complicated because of the frequent absence of validated assays and cutoffs to define antigen positivity in a way that could predict treatment benefit. All these aspects need to be adequately considered when designing trials investigating ADCs.

## Conclusion

ADCs are a potent class of anticancer agents with proved activity across several tumor types. Four of them are already approved, but many others are under preclinical and clinical development. The design of these agents follows the constant progresses in bio-engineering technologies, leading to availability of smarter drugs. Nevertheless, development of ADCs still faces “old” challenges, like biomarker assessment and patient selection, which need to be promptly tackled to exploit this class as its best.

## Data Availability

Not applicable.

## Abbreviations

ADCs: Antibody–drug conjugates
mAb: Monoclonal antibody
ADCC: Antibody-dependent cellular cytotoxicity
CDC: Complement-dependent cytotoxicity
MMAE: Monomethyl auristatin E
MMAF: Monomethyl auristatin F
INN: International Nonproprietary Names
T-DM1: Trastuzumab emtansine
DLTs: Dose-limiting toxicities
TOP1: Topoisomerase I
DX-d: Deruxtecan
T-DXd: Trastuzumab deruxtecan
mBC: Metastatic breast cancer
TPC: Treatment of physician’s choice
PFS: Progression-free survival
OS: Overall survival
FDA: Food and Drug Administration
NSCLC: Non-small cell lung cancer
ORR: Overall response rate
DOR: Duration of response
ILD: Interstitial lung disease
GC/GEJC: Gastric cancer and gastroesophageal junction cancer
SYD985: Trastuzumab duocarmazine
EV: Enfortumab vedotin
mUC: Metastatic urothelial cancer
CR: Complete response
Trop-2: Trophoblast antigen 2
SG: Sacituzumab govitecan
HR: Hormone-receptor
mEC: Metastatic endometrial carcinoma
ICD: Immunogenic cell death
CEACAMs: Carcinoembryonic antigen-related cell adhesion molecules
MTD: Maximum-tolerated dose
LG: Labetuzumab govitecan
NRG: Neuregulins
HER3-DXd: Patritumab deruxtecan
PDX: Patient-derived xenograft
ICIs: Immune-checkpoint inhibitors
HGFR: Hepatocyte growth factor receptor
TKIs: Tyrosine-kinase inhibitors
Teliso-V: Telisotuzumab vedotin
FRα: Folate receptor alpha
GPI: Glycosyl-phosphatidylinositol
MS: Mirvetuximab soravtansine
EOC: Epithelial ovarian cancer
TF: Tissue factor
TV: Tisotumab vedotin
HuMax-TF: Humanized anti-TF mAb
TME: Tumor microenvironment
CSC: Cancer stem cells
DLL3: Delta-like ligand 3
PSMA: Prostate specific membrane antigens
